# To Our Contributors

**Published:** 1877-08

**Authors:** 


### Editorial.

### TO OUR CONTRIBUTORS.

The most important and useful feature of medical journals is the record of cases and the plans of treatment adopted throughout the country, in the various forms of disease that occur. The busy practitioner often neglects to report such as would add greatly to the cause of science, for want of leisure to do so in a manner satisfactory to himself; and thus valuable facts are lost to the profession. Short communications containing a single, though important fact, are considered unworthy of publication, and thus useful truths are only treasured in the mind for the discoverer's especial benefit.

In this way journalists fail to afford that which is most important to their readers. Lengthy theoretical articles, it is true, are read and enjoyed by many members of the profession, and we are always pleased to publish the results of scientific research. We think it requires, however, communications on various practical topics, also, to make a medical journal what it should be.

Our department of Society Reports contains various theoretical and practical discussions, making an important feature for the practicing physician. Now we ask our readers—those who are not regular contributors—to give us an occasional communication, in any form and on any subject they may deem proper.

While such names as LeHardy, Banks, Doughty, Logan, Love, Taliaferro, and others, regular contributors, continue their contributions, we feel assured that our readers will not fail to be interested, yet we always have a vacant page for correspondents who will favor us with something new. We occasionally receive such from distant and neighboring States, as well as various sections of our own, and the interest in and usefulness of our journal would be greatly increased by cutting down the department of selections for the insertion of more such than we are now receiving.

Epidemics of various fatal diseases prevail occasionally throughout the country, such for instance as, meningitis, diphtheria, etc., in the treatment of which physicians differ materially. Nothing is more useful practically, than the publication of plans of treatment and statistics of mortality, and we earnestly solicit them from our readers.

We make the conduct of this journal a labor of love, and not pecuniary profit, and hope it will be made the medium of communications to the profession from many who have not heretofore felt it a duty to write.

